# How to Elicit Thought Disorder During Clinical Interviews: A Practical Guide

**DOI:** 10.1192/bja.2026.10213

**Published:** 2026-05-28

**Authors:** Lena Palaniyappan, Oliver Delgaram-Nejad, Eric YH Chen

**Affiliations:** 1 Douglas Mental Health University Institute, Department of Psychiatry, McGill University, Montreal, Quebec, Canada; 2 Department of Psychology, McGill University, Montreal, Quebec, Canada; 3 Department of Psychiatry, Western University, London, Ontario, Canada; 4 Independent Researcher, the DIALOG project, Exeter, United Kingdom; 5 Orygen, the National Center of Excellence in Youth Mental Health, Melbourne University, Melbourne, Australia; 6 Centre for Youth Mental Health, The University of Melbourne, Melbourne, Australia; 7 Department of Psychiatry, The University of Hong Kong

## Abstract

The reliable assessment of Formal Thought Disorder (FTD), a collection of observable signs of suspected thinking dysfunction, is the cornerstone of interviewing a person with psychosis. A significant gap persists between the success of reliable assessments reported in published rating scales of FTD and the practical constraints of a clinical interview. Validated rating scales largely focus on operationalized criteria for describing items; the procedural aspects of how to elicit these signs often remain implicit. We synthesize the principles and procedures of FTD assessment embedded in rating scales, translating their design into a framework for routine clinical use. Systematic employment of several manoeuvres can help elicit FTD signs: conversational stimulation, cognitive loading, social modulation, and pointed clarifications to stress-test thought, language and communication processes. By making these procedures explicit, we aim to equip clinicians and trainees with a strategic, measurement theory-informed approach to the psychiatric interview when assessing for FTD.

## Introduction

*“I am the finest professorship…This is again the highest activity–double–twenty-five francs–I am double polytechnic irretrievable.”* **– Babette S, in Jung’s Psychology of Dementia Praecox**(Woods, 2011)

*“I love Russian songs. I love Russian speech. I know many Russians who are not Russians, because they speak a foreign language. I know that a Russian is a man who loves Russia. I love Russia. I love France. I love England. I love America. I love Switzerland, I love Spain, I love Italy, I love Japan, I love Australia, I love China, I love Africa, I love the Transvaal. I want to love everybody, and therefore I am God. I am not a Russian and not a Pole. I am a man, not a foreigner and not a cosmopolite. I love the Russian land. I will build a dam in Russia. I know that the Poles will curse me. I understand Gogol because he loved Russia.”* – The Diary of Vaslav Nijinsky(Nijinsky, 2006)

*“You know*… *I*… *when I came to England at eleven 102 road, I went to infant school at the time. I was eleven be tweven in December because you know, was still, I was so tiny for my age // … I’m them girl in Eastenders… Julie crabs’ skin and bone and starvation… I don’t know… school with that them*… *them people somehow, they look and the girl face look and I look like that…”* **-Participant 26AR16, the Discussing Abstract Ideas in Schizophrenia Corpus (DAIS-C)**(Delgaram-Nejad *et al*., 2023)

Spontaneous segments of speech like those above are rare in routine clinical practice. Yet these are the tell-tale signs of Formal Thought Disorder (FTD) – a disruption in thought/language structure and coherence expressed through speech, that represents a core diagnostic feature of severe mental illnesses(Kircher *et al*., 2018). The central question facing time-pressed clinicians looking for FTD is: how can we increase the likelihood of eliciting such revealing speech patterns in the clinic?

The challenge of assessing FTD is fundamentally different from assessing many other psychiatric phenomena. Unlike hallucinations, depression or anxiety, which patients descriptively report, FTD must be actively drawn out to be observed during the clinical interview(Palaniyappan, Homan and Alonso, 2022). This requires clinicians to serve as *architects of conversational conditions* that reveal latent cognitive-linguistic vulnerabilities.

Over decades, several rating scales have demonstrated that FTD can be measured with high inter-rater reliability when specific elicitation procedures are followed(Voppel *et al*., 2025). The authors of these rating scales(Singer and Wynne, 1965; Reilly *et al*., 1975; Johnston, 1979; Andreasen NC, 1979; Harrow and Quinlan, 1985; Caplan *et al*., 1989; Docherty, DeRosa and Andreasen, 1996; Chen *et al*., 1996; Liddle *et al*., 2002; Kircher *et al*., 2014) universally acknowledge that standardization of these procedures is essential to achieve good inter-rater reliability. Yet a paradox persists: despite their psychometric reliability, these scales remain unused in routine clinical practice(Zamperoni, Tan, Sumner, *et al*., 2024). FTD remains what Parnas and Sass(Sass and Parnas, 2017) described as “a grossly neglected concept,” with many psychiatrists in early career stages unfamiliar with its assessment.

Multiple factors have contributed to this decline, including the bewildering array of overlapping terms (e.g., circumstantiality, tangentiality, and derailment) used across scales(Sreeraj *et al*., 2025). While theoretical clarification of FTD phenomenology and definitional ambiguities lies beyond our scope (see (Passby and Broome, 2017; Sreeraj *et al*., 2025)), we address a more practical gap: bringing forth the reliable but implicit elicitation procedures exploited by these scales (Table 1) to regular clinical use. By extracting these methods, we make explicit a core set of conversational manoeuvres that can be synthesized and flexibly deployed in routine interviews. Our aim is to ground clinical artistry in measurement methodology, providing a practical manual for the FTD-focused interview (Box 1). We do not provide an exhaustive checklist of FTD items; for this, we refer readers to the appendix and supplement of another work(Sreeraj *et al*., 2025).

### Core Conceptual Framework

The foundational insight for FTD assessment is deceptively simple: FTD comprises of subtle but *observable* signs, not *reported* symptoms. This distinction has profound methodological implications. Just as eliciting a cardiac murmur requires dynamic auscultatory manoeuvres (e.g., handgrip, Valsalva, squatting, breath holding or a treadmill stress(Kurtz, 1984)), detecting FTD demands deliberate conversational techniques. Without proper technique, genuine pathology may go undetected, while improper technique may generate false positives.

**Figure 1:**
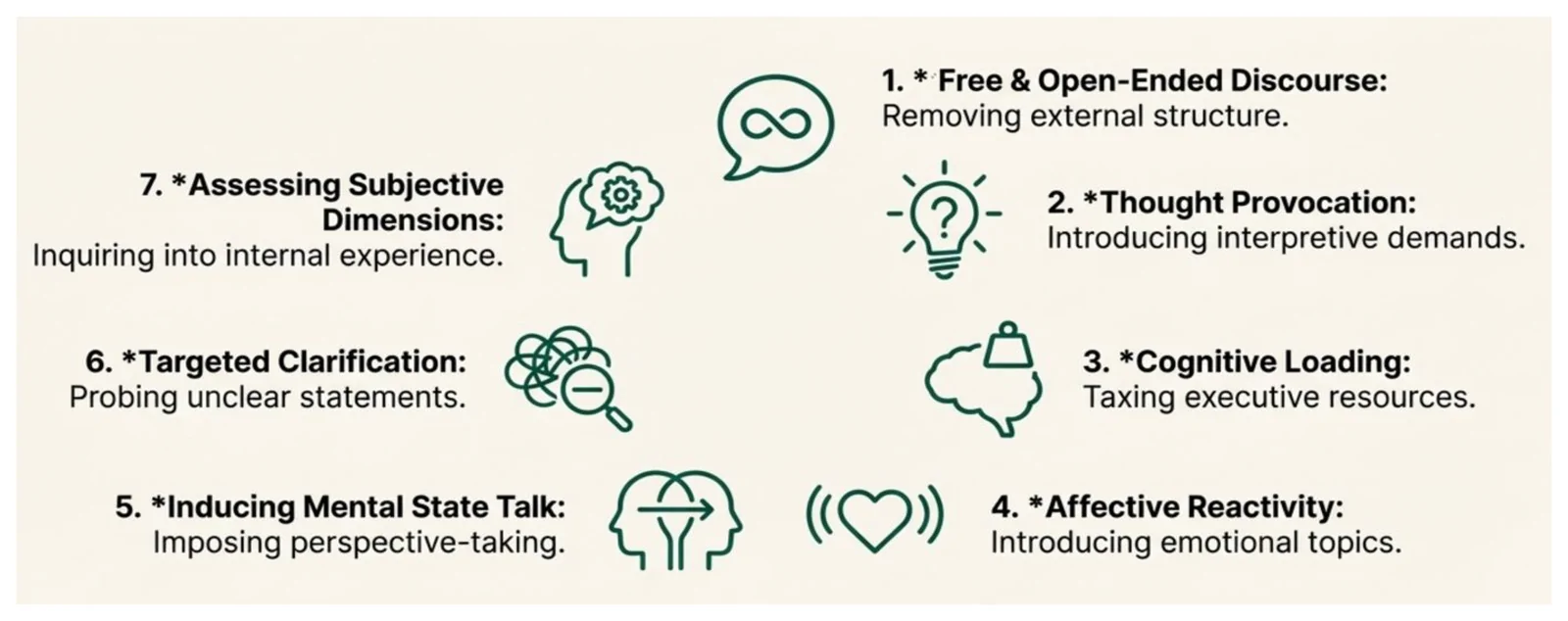
Techniques to elicit Formal Thought Disorder. Starting with free, open-ended discourse, conversational manoeuvres such as using prompts to induce thoughts, increasing processing load via recall demands or emotional content, and inducing social complexity to elicit mental state talk can help a clinician elicit FTD. This figure was generated using Google’s NotebookLM.

Since FTD manifests through conversational speech, our elicitation procedures must themselves be conversational. The clinician becomes an active architect of discourse conditions, strategically varying communicative demands to stress-test cognitive-linguistic systems. Rating scale developers realised early that standard symptom-focused interviews are often insufficient. Patients may only provide brief utterances, without enough linguistic material to assess derailment, loosening, or other phenomena. Successful scales solve this by systematically manipulating discourse conditions through seven key manoeuvres (Figure 1 and Box 2). Employing these manoeuvres provides us with a treadmill test for thought, language, and communication processes.

### Seven Key Manoeuvres for Eliciting FTD

#### 1 Open-Ended Discourse

The rationale for open-ended discourse draws from a fundamental principle: reducing external guides for organising thoughts allows internal organizational deficits (i.e., mental disorganisation that constitutes FTD) to surface. As social dialogue imposes structure through topic cues, probes, and joint attending, external support for organised discourse is optimal. Minimally directed speech (soliloquies) strips away this scaffolding.

Associationist and psychodynamic theories that formed the basis of ‘free association’ technique i.e., saying whatever that comes to mind freely or induced by a trigger word, have fallen from theoretical favour. But given the importance of observing aberrant semantic associations(Barrera, McKENNA and Berrios, 2005) and first-person narratives in FTD(Elleuch *et al*., 2024), a modified form of this approach continues to be important for our purpose. Features such as derailment and perseveration emerge only when patients generate extended linguistic sequences with minimal external constraint. Phenomena like poverty of speech and increased latency become quantifiable through the amount, pacing, and pausing of spontaneous output.

Instead of seeking complete free association of Freudian type, contemporary scales operationalize this principle through open-ended prompts. The Bizarre-Idiosyncratic Thinking(Harrow and Quinlan, 1985) (BIT) scale makes this explicit: "more open-ended procedures better lend themselves to eliciting thought-disordered speech." The Schizophrenia Communication Disorders scale(Bazin *et al*., 2005) (SCD) focuses on everyday life topics – hobbies, family, television – that should elicit natural, elaborated responses. Some authors have resorted to the topic of dreams(Mota *et al*., 2014). Clinical Language Disorder Rating Scale(Chen *et al*., 1996) (CLANG) recommends neutral topics unrelated to psychopathology for this aspect of interview.

#### Clinical Application

Initiate the interview with 5-7 minutes of minimally constrained discourse, asking the patient to speak freely, without checking your response. A prompt: "Tell me about your favourite TV show", or “Thinking back, can you tell me a story about something important that happened to you in your life?”. The key is establishing permissive conditions to initiate the free-flowing thoughts with first-person perspective.

### 2 Thought Provocation Through Stimuli

If open-ended discourse removes external constraints, thought provocation actively introduces interpretive demands. This procedure coincided the shift from free association to projective techniques in the pre-DSM-III era, as ambiguous stimuli were seen as amplifying subtle cognitive aberrations. By requiring the patient to impose meaning on ambiguity, we can stress-test the integrity of conceptual organization.

FTD scales operationalize this through a variety of complex stimuli with varying degrees of ambiguity in visual and auditory modalities. At the most ambiguous end, Rorschach inkblots (Thought Disorders Index(Johnston, 1979) (TDI), Communication Deviance Test(Singer and Wynne, 1965) (CDT)) offer minimal perceptual structure. The Thematic Apperception Test (Murray, 1943) used by Thought & Language Index (TLI)(Liddle *et al*., 2002) and CLANG provides recognizable scenes but leaves narrative connections unstated. Recorded stories (Kiddie-Formal Thought Disorder Scale(Caplan *et al*., 1989) (KFTDS)) present less ambiguous content but require interpretive commentary.

Simple verbal provocation offers a more practical clinical approach. Abstract questions – "Why do you think some people believe in God?" (CLANG) – demand sustained, organized argument. Proverb interpretation (CLANG, BIT) and comprehension items from Weschler Adult Intelligence Scale(*Wechsler Adult Intelligence Scale--Fourth Edition*, no date) used by TDI and BIT ("Why does land in the city cost more than land in the country?") assess the ability to extract and articulate abstract meaning via monologues of extended utterances.

These tasks share a common feature: they require the patient to generate and sustain a coherent response to an interpretive challenge induced at abstract or metaphorical levels on neutral topics that a casual open-ended conversation might conceal. While less abstract topics (e.g., “What would someone have to do if they wanted to smoke a cigarette/wash their hair/do their laundry?(Just *et al*., 2023)”, or “tell me the story of Cinderella(Fradkin *et al*., 2024)” can also serve as a prompt, abstract topics perform better in identifying features of FTD, without the need to wait for a full story to unfold.

#### Clinical Application

Deploy prompts requiring extended interpretive responses in standard mental state examination tests: “What does it mean to say too many cooks spoil the broth?”. Follow with non-directive encouragement if verbal output is insufficient e.g., "Can you say more?". Include complex visual stimuli (preferably featuring human figures as in TAT; see section 5 below for rationale) to provide interpretive challenge.

### 3 Cognitive Loading

Executive dysfunction figures prominently in theoretical models of FTD, with cognitive disorganisation or dyscontrol representing a major factor across multiple analyses(Roche *et al*., 2015; Zamperoni, Tan, Rossell, *et al*., 2024). Yet surprisingly few scales explicitly tax executive resources during assessment.

The SCD takes the most direct approach, embedding deliberate semantic ambiguity into prompts. Polysemic words such as tie, pumps and rags appear in contexts that activate multiple meanings, forcing executive systems to maintain appropriate semantic selection while suppressing competing interpretations. This dual-task demand can unmask subtle FTD. Of note, ambiguity can also increase the likelihood of expressing delusional ideas (for example, see examples from the DAIS-C corpus(Delgaram-Nejad *et al*., 2023)); this has been the basis of Rorschach inkblots and other projective tests. Several models argue that delusions are formed to explain an experienced ambiguity(Denecke *et al*., 2024).

An alternative approach which introduces memory load was originally used by Bleuler (the story of a donkey that soaked its cotton load from Aesop’s fables(Bleuler, 1950)); this is also utilised by contemporary scales like CLANG. Story recall taxes memory, executive control, and narrative organization simultaneously. This differs fundamentally from using stories as thought-provoking stimuli (as in KFTDS), as the cognitive demand of recall creates the stress test, likely taxing the processes required for coherent retelling.

#### Clinical Application

Ask the patient to read an 8-10 sentence short story and request immediate retelling. The resulting discourse reveals how cognitive demands tax organisation of thoughts.

### 4 Affective Reactivity and Emotional Topics

Some people with psychosis show more pronounced symptoms when negative affect is aroused. This thought-affect interface has been observed for features of FTD(Docherty *et al*., 1994; Cohen and Docherty, 2004). Some scales formalise this observation by deliberately introducing emotional arousal as a probe; e.g., Thought & Language Dysfunction Scale(Kircher *et al*., 2014) (TALD) states "Since some phenomena occur solely under situational stress (delusions, hallucinations, emotional life events), emotional topics should be raised." This procedure operates under the assumption that if capacity to organise thoughts is marginal, emotional arousal may precipitate decompensation into manifest FTD. For some, describing delusional or hallucinatory content may be the only context where FTD becomes manifest.

Of note, many healthy individuals show transient speech disorganisation when intensely emotional– a phenomenon that forms the basis for Main’s Adult Attachment Interview to identify disorganized attachment(George, Main and Kaplan, 1985). The SCD acknowledges this caveat and takes the approach of advocating ‘shielded’ assessment that avoids discussion of symptoms entirely. This methodological choice also addresses another concern of ‘symptom leakage’ or contamination, e.g., awareness of a patient’s bizarre delusions or hallucinations may bias observers to rate speech as more illogical or loose than it actually is(Palaniyappan, Homan and Alonso, 2022). The ideal solution – though difficult in clinical practice – involves separate assessment contexts: one shielded from symptom content (early part), another deliberately engaging emotional reactivity (later part). Comparing discourse across contexts provides the most informative picture.

#### Clinical Application

After establishing baseline patterns through neutral discourse (the first 3 phases above), gradually introduce personally relevant emotional topics, e.g., health related topics (symptoms or treatment). Monitor for structural deterioration: Does logical sequencing fragment? Do referential connections become unclear? Does coherence collapse under emotional load and then recover?.

### 5 Inducing Mental State Talk

A growing body of literature on social cognition and formal thought disorder indicates that issues with theory of mind (i.e., perspective taking) may explain some of the features of FTD(Hardy-Baylé, Sarfati and Passerieux, 2003; Minor *et al*., 2015; Achim, Achim and Fossard, 2022). The closest approximation to testing this aspect appears in the SCD’s assessment of perspective-taking: "In your opinion, what does your father, mother, friend, doctor think about your problems?" This requires modelling others’ mental states – a social-cognitive demand revealed in ‘mental state talk(Kim *et al*., 2021)’ (e.g., "*She knows that he is going to leave her")*. Picture description tasks featuring human figures also involve implicit social demand modulation. Describing what characters are doing, thinking, or feeling requires theory of mind operations that engage social cognition alongside language organization. Furthermore, the need to generate syntactically more complex clauses (e.g., when describing who did what to whom) in stories, also increases the likelihood of grammatical errors associated with FTD(Elleuch *et al*., 2025). The integration of these demands can unmask deficits not apparent in non-social narratives.

#### Clinical Application

Impose perspective-taking demands: "If I asked your colleague/best friend about you, what would they say?" This requires adopting another’s perspective while maintaining narrative coherence. Use of TAT pictures with human characters or cartoon storyboards(Achim, Achim and Fossard, 2022; Alonso-Sánchez *et al*., 2022; Cho *et al*., 2025) may also enforce perspective taking and mental-state talk.

### 6 Targeted Clarification

Of all the techniques derived from rating scales, seeking targeted clarification is one the most important for clinical judgement. When explicitly requesting clarification for an unclear or illogical statement, the patient’s response helps us to differentiate accidental slips or drifts (successfully clarified) from more pervasive thought disorder (further confabulation or tangentiality). This should be practiced at all phases of the interview.

Many factors can produce unclear speech – anxiety, unfamiliarity with medical settings, limited education, or simply the universal difficulty of converting complex thoughts into linear language. Seeking clarification involves asking patients to access and re-present the thought they just expressed. If the thought is intact but expression was poor, clarification succeeds. If the thought is disorganised, clarification may reveal further drift. Successful clarification after prompts indicates effective conversational scaffolding; the ability to repair unclear speech with clinician support may suggest milder formal thought disorder compared to cases where scaffolding fails to improve coherence.

Multiple scales incorporate this principle. TDI instructs raters to "inquire into all deviant verbalizations, percepts, slips, and reasoning." BIT requires inquiry into "all unclear or odd words or responses.” TLI structures this as systematic follow-up after picture descriptions. SCD goes a step further and marks “the inability to clarify a speech corpus” as a marker of deficit in the integration of contextual information. The consistency across scales reflects recognition that clarification requests are not optional add-ons but essential diagnostic manoeuvres.

#### Clinical Application

When an unclear or illogical statement occurs, resist the urge to move on. Instead ask: "I want to make sure I understand – what did you mean by that?" or "Can you explain that in a different way?" Successful clarification weakens the case for FTD. Progressive confusion, tangential drift, or bizarre elaboration strengthens it.

### 7 Subjective Dimensions

Formal thought disorder encompasses both observable signs and subjective experiences. Observable signs are the external manifestations of disrupted thought organisation, including disturbances in the structure of speech (e.g., derailment, tangentiality, incoherence) and in its dynamics (e.g., pressure of thought, flight of ideas); these are what clinicians directly observe, and what FTD rating scales such as the TLC(Andreasen NC, 1979) and TALD(Kircher *et al*., 2014) are designed to capture. Subjective experiences are patients’ first-person awareness of their own thinking processes, such as thoughts that feel blocked, racing, or out of control(Kircher *et al*., 2014). These two dimensions do not always correlate perfectly(Kircher *et al*., 2014): many patients with profound objective disorganisation report minimal subjective awareness. Direct questions about patients’ internal experiences therefore complement, rather than substitute for, observational techniques (Table 2). Notably, subjective features of thought-language organisational dysfunction may phenomenologically overlap with cognitive/psychomotor slowing of depressive episodes(Palaniyappan and Wang, 2025) with common elicitation procedures as we describe here. A list of these, adapted from TALD(Kircher *et al*., 2014), is provided in Table 2 for clinical application.

#### Practical Considerations and Limitations

**Figure 2:**
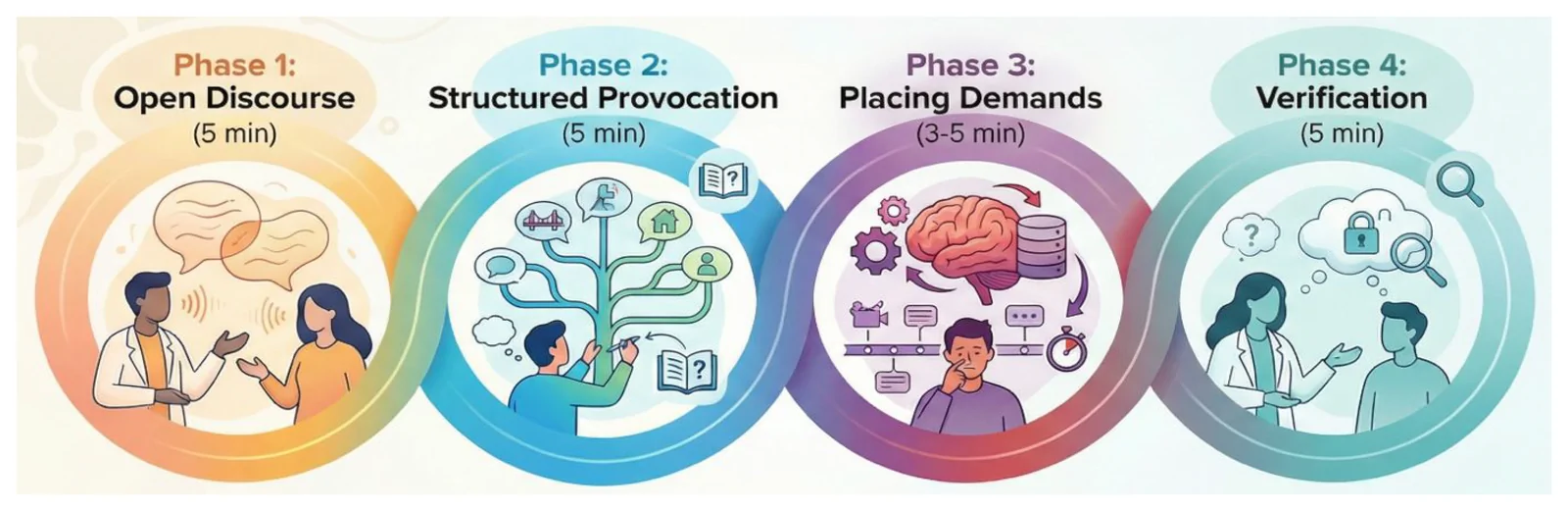
An Integrated Clinical Approach to elicit Formal Thought Disorder. A sensitive clinical assessment of FTD needs to follow a structured, phased approach: **Phase 1: Open Discourse (5 minutes):** Start with minimal restrictions to establish baseline patterns. Ask open-ended questions about neutral topics (hobbies, daily life, interests) to encourage natural speech without structure. This phase shows spontaneous organisation, speech quantity and pacing, and baseline coherence before introducing more demands. **Phase 2: Structured Provocation (5 minutes):** Introduce interpretive and narrative demands for more goal-directed thoughts. Story generation, proverb interpretation, abstract questions, and complex picture descriptions all need organising around external prompts. This phase shows how organisational processes work when needed for specific communication goals. **Phase 3: Placing Demands (3-5 minutes):** Deliberately stress cognitive-linguistic systems. Story recall increases memory load. Emotional content engages the thought-affect interface. Inducing mental state talk via perspective-taking taxes social cognition. Not all patients need this phase (e.g., someone with pressured speech in a manic episode) – but when earlier phases yield ambiguous results, stress-testing can be crucial. **Phase 4: Verification of subjective phenomena (5 mins):** Finally enquire about subjective aspects of thought disorder. Throughout all phases of the interview, systematically seek **clarification** for any deviations, unclear statements, or illogical connections noted. This figure was generated using Google’s NotebookLM.

It is important to note that not all seven elicitation manoeuvres (structured across 4 phases in Figure 2 for ease of administration) are equally specific for diagnosing FTD. Findings from affective provocation should be interpreted cautiously. Transient disorganisation can occur in healthy individuals under anxiety or strong emotion, in patients with limited formal education or familiarity with clinical settings, and in those speaking a second language. Spontaneous disorganisation (phase 1 and 2) that worsens with cognitive loading or perspective-taking demands (phase 3), and disorganisation that cannot be repaired through targeted clarification (phase 4), provide stronger evidence for genuine FTD. Clinicians should seek convergent evidence across multiple manoeuvres and prioritise recurring instances over isolated findings from the various phases of the interview. Broader controversies and ongoing debates regarding interpretation and diagnostic thresholds are summarised in Box 3.

#### Time Constraints

Starting with free, open-ended discourse, followed by visual and abstract prompts to induce thoughts, increasing processing load via recall demands or emotional content, and finally inducing social complexity to elicit mental state talk provides an optimal set of approaches to elicit FTD in a clinical setting. While there are several other approaches (e.g., showing videos of emotional stimuli and then asking standardized questions(Huang *et al*., 2026)), clinical reality often complicates idealized assessment procedures. Patients with negative symptoms may lack the motivation for extended discourse. Guarded patients may resist procedures perceived as testing their sanity. Children may find formal assessment threatening. Assessing FTD in a gamified manner with picture descriptions, story-telling and collaborative exploration as laid out above, or with playful word associations (that help assess semantic priming) shifts the focus from evaluation to shared activity with the clinician. Such gamification can reduce the Hawthorne effect on verbal behaviour and ensure engagement for the 15-20 minutes of discourse needed for a reliable assessment.

FTD may manifest in written material (letters, texts, emails) and does not always co-occur with spoken FTD. Clinicians should review written samples when available, though care is needed when interpreting FTD in text messaging due to format-specific conventions.

#### Significant others

A growing body of evidence from individuals with lived experience of FTD highlights a crucial yet often overlooked dimension: the listener’s role in co-constructing what clinicians call disordered speech(Galletly and Crichton, 2011; Hodgins, O’Driscoll and Titone, 2022). Communication is inherently dyadic; what one person finds incoherent may be understood by another. This insight challenges assumptions about the objectivity of FTD assessment and highlights the value of underutilised assessment strategies, such as systematic observation of conversations with others (third parties). This faces practical constraints in a clinical setting. Enquiring about conversational difficulties encountered by people who spend considerable time with the patient will be a valuable source of information.

#### Cultural and Linguistic Considerations

Assessing FTD across different cultural and linguistic contexts requires additional care. What appears as loose associations or circumstantiality may reflect cultural narrative styles or translation difficulties. Some languages have features that complicate assessment; For instance, Tamil has no formal definite (the) or indefinite (a/an) articles but relies on context and demonstratives. A native Tamil speaker may miss such nuances in English, and this needs to be accommodated in our assessments. Another example is Cantonese, where the same phonetic sound can map onto multiple words; these frequent homophones and puns should not be considered pathological. Whenever possible, involve clinicians familiar with the patient’s cultural background and primary language. When using interpreters, ensure they understand the assessment goals and can convey not just content but also the structure and coherence of the patient’s speech.

#### Documentation

Detailed documentation should specify the discourse context in which FTD was observed or absent (neutral-concrete, neutral-abstract, emotional, psychosis-related topics). Record verbatim examples whenever possible (e.g., using embedded dictation systems) including the elicitation method used (e.g., picture description, proverb interpretation) and whether clarification attempts were successful. This provides a richer source for longitudinal comparison, assess treatment effects and allows other clinicians to understand the basis for your assessment.

#### Implications for Training

The impression of decline in FTD assessment skills among psychiatrists(Tucker, 1998; Sass and Parnas, 2017) reflects, in part, inadequate training(Zamperoni, Tan, Sumner, *et al*., 2024). Traditional teaching often portrays FTD as a passive observation rather than an active elicitation skill. Medical students and trainees can be explicitly taught these manoeuvres, with practice and feedback opportunities. Reviewing clinical interview recordings and discussing them provides valuable learning. Transcribing interview portions helps analyse speech patterns and sharpens observational skills. Workplace-based assessments could evaluate trainees’ ability to elicit FTD using these techniques. Exposure to validated rating scales, even if not used clinically, provides frameworks for understanding FTD phenomenology. The publicly available DAIS-C(Delgaram-Nejad *et al*., 2023) schizophrenia speech corpus, including participants with advanced FTD, and the interviewer being a lived experience expert(Delgaram-Nejad, 2025, no date) can be a useful training aid for clinicians reviewing recent interactions. FTD per se is not a single natural kind; it is a clinical entity constituted for practical purposes(Palaniyappan *et al*., 2026). Sharpening our training to reliably elicit all its constituents is key to its utility in the psychiatric clinic.

## Conclusion

Babette’s "double polytechnic irretrievable", Nijinsky’s stream of disconnected statements and the more recent example from participant 26AR16 from DAIS-C, all represent the dramatic end of the FTD spectrum. But FTD exists along a continuum, from subtle loosening detectable only through systematic probing to profound disorganization that overwhelms all communication. Mastering the methodological skills of stress-testing, clarification and verification of subjective experience of FTD will improve how we measure, document, and understand thought disorder as it unfolds in front of us at our clinics.

## Supplementary Material

Supplementary Material

## Figures and Tables

**Table 1:** Overview of Major FTD Rating Scales and their Elicitation Procedures

Scale (Acronym; Year)	Key Stimuli & Tasks
Wynne and Singer’s Communication Deviance Test (CDT; 1963)	Viewing and responding to TAT and Rorschach cards; corresponds to everyday situations in which "two people see a series of different kinds of reality."
Reilly’s Loosening of Association and Disordered Speech (LADSP; 1975)	Free verbalization of non-mental health topic (7 mins) and mental health topic (7 mins) to induce potential stress, with prompts only to encourage flow.
Andreasen’s Thought, Language & Communication Scale (TLC; 1979)	Open interview on neutral and standard clinical topicsa. Two phases: uninterrupted speech, then introduce some interruptions.
Holzman & Johnston’s Thought Disorder Index (TDI; 1979)	Rorschach Inkblots; WAIS subtests (Comprehension, Similarities). All deviant verbalization inquired.
Harrow’s Bizarre-Idiosyncratic Thinking Index (BIT; 1985)	Gorham Proverbs Test and WAIS Comprehension Subtest with inquiry into odd responses.
Caplan’s Kiddie-FTDS (K-FTDS; 1989)	Story Game: Retelling audio stories and generating original stories from prompts. Non-directive prompts during the game (e.g., "Can you say more?").
Docherty’s Communication Deviance Index (CDI; 1996)	Open-ended questions on neutral topics (family, religion, politics). No active provocation.
Chen’s Clinical Language Disorder Rating Scale (CLANG; 1996)	Combination of spontaneous speech, open questions (e.g., "Why believe in God?"), cognitive tests, narrative tasks.
Liddle’s Thought & Language Index (TLI; 2002)	Sequential description of 8 TAT pictures (1 min each), followed by structured inquiry for clarifications and to follow logic of thought.
Hardy-Baylé & Bazin’s Schizophrenia Communication Disorders Scale (SCD; 2005)	Adversarial Interview with specific constrained tasks (e.g., resolve ambiguity after a distractor math task) where clinician deliberately creates conversational challenges.
Kircher’s Thought & Language Dysfunction Scale (TALD; 2014)	50-min exploration. Open-ended start, then semi-structured clinical questions. Emotional topics (around delusions) deliberately raised and subjective aspects directly questioned.
WAIS: Wechsler Adult Intelligence Scale; TAT thematic perception task. a. While Andreasen described a 50-min interview for scoring TLC, researchers have applied numerous variations ranging from a set of 6 questions, a 5-10 minutes interview(Muralidharan *et al.*, 2018), to using very recordings of free speech (~1.5–3 min)(Tang *et al.*, 2023) or snippets from videos of ~1.9m average length(Krell *et al.*, 2022)	

WAIS: Wechsler Adult Intelligence Scale; TAT thematic perception task. a. While Andreasen described a 50-min interview for scoring TLC, researchers have applied numerous variations ranging from a set of 6 questions, a 5-10 minutes interview(Muralidharan *et al*., 2018), to using very recordings of free speech (~1.5–3 min)(Tang *et al*., 2023) or snippets from videos of ~1.9m average length(Krell *et al*., 2022)

**Table 2:** Interview Questions for Subjective aspects of FTD

Interview Questions
1. **Initiative**: Is thinking too much of an effort for you these days? Do you feel like you have the mental energy you used to have?
2. **Slowing**: Does your thinking feel slowed down, as if it is a heavy drag?
3. **Blocking**/**Fading**: Does your train of thought ever just suddenly stop mid-sentence, like hitting a wall? Do your thoughts sometimes just slowly fade away?
4. **Poverty**: Have you noticed fewer new ideas or thoughts coming to you than before?
5. **Pressure**: Have you experienced too many thoughts coming at once, like you can’t control them?
6. **Interference**: Do you find it hard to stay focused because other thoughts keep jumping in, or things around you keep pulling your attention away?
7. **Rumination**: Are there certain thoughts or worries that you can’t seem to stop thinking about, even when you try to move on to something else?
8. **Reception**: When people are talking to you, can you follow along and understand them as easily as before? Do you find some words or sentences confusing?
9. **Expression**: How about expressing yourself - do you feel like you can say what you mean as clearly as you used to? Do you find some words or phrases had to come by?

Questions are organized in a natural conversational flow, starting with energy, speed and moving through specific domains of thinking, attention, and communication. These subjective items are modified from Kircher’s Thought and Language Disorder Scale.

## Box 1 Key Learning Points

- Formal Thought Disorder (FTD) is a collection of observable signs that must be actively elicited through systematic conversational manoeuvres, not passively awaited.
- Seven key elicitation techniques derived from validated rating scales include: free association, thought provocation, cognitive loading, affective reactivity, manipulation of perspectives, systematic clarification, and gamification.
- A structured, phased approach to FTD assessment integrates these techniques across 15-20 minutes: open discourse, structured provocation and social-cognitive-emotional challenge.
- The clarification technique is critical for differentiating occasional slips from genuine thought disorder – patients without FTD can usually clarify their meaning or repair inconsistencies when prompted.
- Cultural context, linguistic factors, and differential diagnoses (aphasia, intellectual disability, anxiety) must be carefully considered to avoid misidentifying normal variations or other conditions as FTD.

## Box 2 Good Practice Points

- Allocate at least 15 minutes for dedicated FTD assessment, beginning with neutral, open-ended topics before progressing to more demanding tasks.
- Systematically request clarification for any unclear, illogical, or tangential statements, and verify if there are subjective experiences of disrupted thought flow and dynamics
- When working with interpreters, brief them on the assessment goals and ensure they can convey speech structure and coherence, not just content.
- Balance assessment of observable FTD signs with direct inquiry about the patient’s subjective experience of their thinking processes.
- With patient’s consent, consider recording verbatim portions of clinical interviews for more detailed analysis rather than simply noting "disorganized speech - present or absent."

## Box 3 Controversial Issues and Ongoing Debates

- The theoretical basis of FTD remains debated: is it primarily a disorder of associations, executive control, semantic memory, or communication pragmatics?
- The specificity of FTD to psychotic disorders is questioned, as similar phenomena occur in mania, dementia, and even normal individuals under stress or fatigue.
- Whether FTD should be understood as a categorical sign (present/absent) or a dimensional phenomenon existing on a continuum remains unresolved.
- The role of the listener in identifying speech as "disordered" is increasingly recognized, raising questions about the objectivity of FTD assessment.
- Emerging computational linguistics and natural language processing approaches may provide objective, automated FTD assessment, but their clinical utility and ecological validity remain to be established.
- The relationship between observable FTD and subjective thought disorder experiences (racing thoughts, thought blocking) is complex and not fully understood.

## Learning Objectives

*After reading this article you will be able to:*

1. Understand the conceptual framework for Formal Thought Disorder (FTD) as a collection of observable signs requiring specific elicitation procedures
2. Apply systematic conversational manoeuvres including thought provocation, cognitive loading, and inducing mental state talk to reliably elicit FTD signs during clinical interviews
3. Implement a structured, phased approach to FTD assessment that integrates evidence-based techniques from validated rating scales into routine clinical practice
