# Supplementary material for: How to Elicit Thought Disorder During Clinical Interviews: A Practical Guide
Source: BJPsych Adv. Author manuscript; Available in PMC 2026 Nov 2. (PMC7619686; doi:10.1192/bja.2026.10213)
Supplement: Supplementary file 1 [file EMS213391-supplement-Supplementary_Material.pdf]

Multiple Choice Questions

1. Which of the following best describes the conceptual basis for assessing Formal Thought Disorder (FTD)?

a) FTD is a subjective symptom that patients will spontaneously report if present
b) FTD is a collection of observable signs that requires active elicitation
c) FTD can only be reliably assessed using standardized rating scales
d) FTD assessment should focus exclusively on the patient's written communication
e) FTD is best evaluated through passive observation during routine clinical conversation

Answer: b
Explanation: FTD is a collection of observable signs requiring active elicitation, not passive observation. Patients do not typically complain of FTD, and the clinician must use specific conversational techniques to reveal latent thinking dysfunction.

2. What is the primary purpose of requesting clarification when a patient makes an unclear or illogical statement?

a) To demonstrate to the patient that their communication is problematic
b) To fill time during the interview when uncertain how to proceed
c) To differentiate between poor expression and genuine thought disorder
d) To test the patient's frustration tolerance and emotional regulation
e) To allow the interviewer time to formulate their diagnostic impression

Answer: c
Explanation: Seeking clarification regarding unclear segments of speech helps us distinguish between expression difficulties (where the patient can successfully clarify their meaning) and genuine thought disorder (where clarification attempts produce further confusion or tangential responses).

3. Which of the following represents an appropriate application of cognitive loading in FTD assessment?

a) Asking rapid-fire questions without allowing time for responses
b) Reading a brief story and asking the patient to retell it in their own words
c) Conducting the interview in a noisy environment with multiple distractions
d) Requiring the patient to write their responses rather than speaking to them
e) Repeatedly interrupting the patient mid-sentence to test their frustration tolerance

Answer: b
Explanation: Cognitive loading involves introducing tasks that tax executive function and working memory, such as story recall. This differs from simply creating a distracting environment or interrupting the patient, which would not systematically assess cognitive-linguistic capacity.

4. When assessing FTD across different cultural and linguistic contexts, which approach is most appropriate?

a) Apply standardized Western assessment criteria uniformly across all cultural groups
b) Avoid assessing FTD in non-native speakers as it cannot be done reliably
c) Focus only on non-verbal behaviours when working with interpreters
d) Ensure interpreters can convey speech structure, not just content
e) Rely exclusively on self-report measures to avoid cultural bias

Answer: d
Explanation: Cultural and linguistic considerations require involving clinicians familiar with the patient's background and ensuring interpreters understand the assessment goals and can convey not just content but also the structure and coherence of speech. This balances cultural sensitivity with systematic assessment.

5. The recommended phased approach to FTD assessment typically requires what minimum duration?

a) 3-5 minutes
b) 7-10 minutes
c) 15-20 minutes
d) 30-45 minutes
e) 60 minutes or more

Answer: c
Explanation: We recommend at least 15-20 minutes for dedicated FTD assessment (with many instruments requiring 15 minutes minimum), structured across phases: open discourse, structured provocation, cognitive-emotional challenge with ongoing clarification throughout, along with verification of subjective phenomena.
